# Mindfulness-Based Interventions for Young Offenders: a Scoping Review

**DOI:** 10.1007/s12671-018-0892-5

**Published:** 2018-02-21

**Authors:** Sharon Simpson, Stewart Mercer, Robert Simpson, Maggie Lawrence, Sally Wyke

**Affiliations:** 1 General Practice and Primary Care, Institute of Health and Wellbeing, University of Glasgow, Glasgow, Scotland G12 9LX UK; 2 School of Health and Life Sciences, Glasgow Caledonian University, Glasgow, Scotland G4 0BA UK; 3 College of Social Science, Institute of Health and Wellbeing, University of Glasgow, Glasgow, Scotland UK

**Keywords:** Incarcerated, Mindfulness, Meditation, Offending, Scoping review

## Abstract

Youth offending is a problem worldwide. Young people in the criminal justice system have frequently experienced adverse childhood circumstances, mental health problems, difficulties regulating emotions and poor quality of life. Mindfulness-based interventions can help people manage problems resulting from these experiences, but their usefulness for youth offending populations is not clear. This review evaluated existing evidence for mindfulness-based interventions among such populations. To be included, each study used an intervention with at least one of the three core components of mindfulness-based stress reduction (breath awareness, body awareness, mindful movement) that was delivered to young people in prison or community rehabilitation programs. No restrictions were placed on methods used. Thirteen studies were included: three randomized controlled trials, one controlled trial, three pre-post study designs, three mixed-methods approaches and three qualitative studies. Pooled numbers (*n* = 842) comprised 99% males aged between 14 and 23. Interventions varied so it was not possible to identify an optimal approach in terms of content, dose or intensity. Studies found some improvement in various measures of mental health, self-regulation, problematic behaviour, substance use, quality of life and criminal propensity. In those studies measuring mindfulness, changes did not reach statistical significance. Qualitative studies reported participants feeling less stressed, better able to concentrate, manage emotions and behaviour, improved social skills and that the interventions were acceptable. Generally low study quality limits the generalizability of these findings. Greater clarity on intervention components and robust mixed-methods evaluation would improve clarity of reporting and better guide future youth offending prevention programs.

**Electronic supplementary material:**

The online version of this article (10.1007/s12671-018-0892-5) contains supplementary material, which is available to authorized users.

##  Introduction

Many countries around the world place a high priority on the rehabilitation of young people who offend. This group is typically characterized by socio-economic deprivation, adverse childhood experiences (ACEs), low educational attainment, difficulties with regulating emotions and behaviour, poor mental health and impaired quality of life (QOL) (Dodge and Pettit 2003; Ou and Reynolds 2010). A growing body of evidence suggests that such factors contribute to delayed maturational development and impaired social skills (Monahan et al. 2009, 2013) and have detrimental effects on neural development in brain areas thought important in executing the cognitive control required for regulating emotions (Abram et al. 2004). Difficulties with regulating emotions and behaviour, poor cognitive abilities and coping skills and poor mental health have all been postulated as important determinants of subsequent offending behaviour among young people. The “What Works” report on factors that may reduce re-offending suggests that offenders are more likely “to desist from offending if they manage to acquire a sense of control over their own lives and a more positive outlook on their future prospects” (Sapouna et al. 2011, p. 24). The report suggests that interventions aimed at enhancing coping skills and psychological resilience are most likely to reduce re-offending.

Clearly, the prevention of youth offending by tackling the wider social determinants of health is a much needed upstream solution, which depends on political and societal responses to inequalities (Marmot 2005), but interventions at a group or individual level are also needed to help those currently affected. These young people represent a particularly vulnerable group, and effective interventions are needed to help them manage stress and improve cognitive and emotional skills. Currently, the most researched interventions for those in custody are based on cognitive behavioural therapy (CBT) principles (Andrews et al. 1990; Lipsey 1995; Lösel 1995). Although CBT has strong empirical backing, the evidence remains limited in this specific population and it is recognized that this approach may need supplementation (Sapouna et al. 2011; Ward et al. 2012; Wilson and Yates 2009). Recently, increasing attention has been placed on natural protective factors, individual strengths and positive treatment alliances (Ward et al. 2012). One such approach that may be useful in this regard is mindfulness.

Mindfulness-based interventions (MBIs), derived from Buddhist meditation practices and secularized for use in contemporary society, preferentially train attentional awareness, enhancing emotional and behavioural regulatory skills and generating a shift in one’s perspective of self (Hölzel et al. 2011). MBIs have a growing evidence base for use within clinical and non-clinical settings alike, improving both psychological functioning and wellbeing in people with chronic health problems (Bohlmeijer et al. 2010; Fjorback et al. 2011; Goyal et al. 2014; Mars and Abbey 2010). Systematic reviews, meta-analyses and randomized controlled trials (RCTs) suggest that MBIs may be potentially useful in numerous relevant domains, including the management of anxiety (Grossman et al. 2004; Hofmann et al. 2010), stress (Chiesa and Serretti 2009), depression (Baer 2003; Kuyken et al. 2008; Teasdale et al. 2002), trauma (Kuyken et al. 2015) and addictive behaviours and substance misuse (Witkiewitz et al. 2005, 2013). Further, those studies that have included active comparator groups suggest that, in general, MBIs are as effective at improving mental health and wellbeing as other commonly used interventions in this context, such as CBT or antidepressants (Goyal et al. 2014; Kuyken et al. 2015). In addition, systematic review evidence suggests beneficial effects from manualized MBIs on memory, some aspects of executive function (inhibition and set shifting), cognitive flexibility and meta-awareness (Lao et al. 2016). Based on this evidence, there are several reasons to hypothesize why mindfulness may have particular relevance for young offenders.

A number of studies have demonstrated an association between youth offending and the ability to self-regulate emotions, in particular impulsivity and impaired cognitive and behavioural flexibility (Chitsabesan et al., 2006; Fazel et al. 2008; Vitacco et al. 2002, 2010). Ability to regulate emotions requires effective executive functioning, that is, the ability to inhibit inappropriate behaviour (inhibitory control), activate an appropriate response (activation control) and shift and focus attention as required (effortful control) and to integrate information, plan, detect error and modify behaviour as necessary (Rothbart 2007; Rothbart et al. 2007). Maturational brain changes between adolescence and early adulthood may explain why some young people who offend early on seem to show decreases in impulsivity and improvements in self-control over time (Loeber et al. 2013). During this stage of maturation, risk perception is refined, resistance to peer influence strengthened, anticipation of future consequences improved and sensation seeking and impulsivity lessened (Steinberg 2007; Steinberg et al. 2008, 2009). For those not obviously showing such maturation and improvement, seeking to assist the developmental process through targeted intervention makes sense.

In addition, stress that is perceived as uncontrollable can rapidly impair performance on tasks requiring top-down, prefrontal cognitive control (Arnsten 1999; Cerqueira et al. 2007) and has also been shown to impair self-regulatory ability in adolescents (Duckworth et al. 2013). Exposure to such stressors is thought to direct processing away from higher cognitive functioning, behaviours instead being driven by emotion-based systems associated with increased vigilance and scanning of the environment for the detection of potential threat. This may lead to impairments in self-regulatory processing, where the individual has difficulty with the experiencing, interpreting, regulating and managing of emotional state(s) (DeBellis and Thomas 2003). It seems clear that youth offending populations represent a particularly vulnerable group, with a need for effective interventions that can improve cognitive and emotional skills and the ability to manage stress.

Mindfulness meditation has been shown to strengthen neural pathways between areas of the prefrontal cortex and limbic system associated with regulating the stress response and emotional experience (Hölzel et al. 2011). Mindfulness training is proposed to provide the cognitive tools to deal more skillfully with mental reactions to stressors, and a primary outcome of enhanced mindfulness is the improved capacity for experiencing and tolerating negative affect and distressing emotional states, both common findings among offending populations (Witkiewitz et al. 2013). Thus, MBIs may serve to address several key constructs underlying offending behaviours (Farrington 2000).

As we move towards evidence-based practice in forensic psychology settings, such as young offenders’ institutions, it is important to determine whether novel treatments such as MBIs do constitute potential rehabilitative options for young people involved with the criminal justice system. However, before undertaking further primary evaluations, it is necessary to take stock of existing research evidence in this area. This will help us determine what is already known and available in the literature base regarding the potential benefits of a MBI within youth offending populations. The current review has four objectives: (1) determine the types of studies that have been published regarding the use of MBIs within youth offending populations, (2) identify the population characteristics of the groups in which MBIs have been studied, (3) determine the specific types of intervention strategies that have been used and (4) ascertain what outcomes have been assessed and what they have shown.

## Method

### Search Strategy

This scoping review followed the five-stage approach advocated by Arskey and O’Malley (2005), incorporating the more recent recommendations made by Levac et al. (2010). In October 2016, nine electronic bibliographic databases and information repositories (MEDLINE, PsycINFO, EMBASE, CINAHL, ASSIA, Science Direct, Cochrane Library, Web of Science and Allied and Complementary Medicine Database (AMED)) were searched along with the ProQuest Dissertations & Theses database. Selected subject headings were combined with key words relating to mindfulness and offending to create a search strategy that was finalized for use in MEDLINE and modified as required for use in other databases, using Boolean operators, search symbols and controlled vocabulary. Hand searching of the reference lists of included papers for potentially relevant studies not identified by the database searches was also carried out. Studies were deemed relevant at this stage if the title included one or more of the agreed search terms.

### Selection Criteria

Studies were selected for inclusion if (1) the intervention included at least one of the three main techniques of mindfulness-based stress reduction (MBSR) (i.e. breath awareness, body awareness or mindful movement), as it most closely represents the standardized model; (2) the focus was on youth offending populations (incarcerated or being rehabilitated in the community); and (3) any discernable methods were used (either quantitative or qualitative) to assess primary data.

Studies were excluded if they were non-human, written in a language other than English or published prior to 1980 (after which, MBSR first appeared in the published literature). No restrictions were placed on study design. However, all included studies had to contain primary data (i.e. original research obtained through first hand investigation). Therefore, secondary data sources such as expert opinion papers were not included. No restrictions were in place with regard to study quality. However, in the current review, each of the included studies was quality assessed to assist the reader when interpreting the findings.

### Selection of Papers for Inclusion

Two reviewers (SS and RS) screened titles and abstracts using the inclusion/exclusion criteria to select potentially eligible papers. Copies of the full papers were obtained and authors contacted when necessary to determine whether the intervention met the inclusion criteria. Both reviewers independently read the full papers to determine if they met inclusion criteria. Any discrepancies were adjudicated over by a third, more senior reviewer (SM).

### Quality Appraisal

Quality appraisal was used to assess the quality of the literature (i.e. to determine whether each study was carried out correctly, in line with existing recommendations and standards). It was not intended to stratify papers into a hierarchy of evidence (Daudt et al. 2013; Petticrew & Roberts, 2008). Papers were not excluded from the review on the basis of poor quality methods. For the qualitative studies, a quality appraisal tool based on Spencer et al. (2003) “framework for assessing qualitative evaluations” (FAQE) was used as an aid to provide informed judgment rather than a mechanistic approach. This framework consists of 18 open-ended questions, governed by four guiding principles: (1) contributory (i.e. has knowledge and understanding been extended by this research?), (2) defensible in design (i.e. do the researchers use an appropriate study design to address the research question posed?), (3) rigorous in conduct (i.e. have the researchers been systematic and transparent in their collection, analysis and interpretation of the data?) and (4) credible in claim (i.e. are well-founded and plausible arguments offered?). As this tool does not have an overall rating category, three reviewers (SS, SM and SW) devised one consisting of three categories: (a) strong (*80% or more of the quality indicators were met*), (b) moderate (*between 40 and 80% of the quality indicators were met*) and (c) weak (*less than 40% of the quality indicators were met*).

To assess the quality of the quantitative studies, the “Effective Public Health Practice Project” (EPHPP) quality appraisal tool was used (Armijo-Olivo et al. 2012; Thomas et al. 2004). EPHPP is a 21-item checklist that highlights sources of bias including (1) selection bias, (2) study design, (3) confounders, (4) blinding, (5) data collection methods, (6) withdrawal and dropouts and (7) intervention integrity. The EPHPP tool also allocates a score of strong, moderate or weak rating, but it is important to note that the scores applied are not equivalent across the two systems of criteria used to quality appraise the different studies (i.e. qualitative and quantitative).

The search also identified studies that employed a mixed-methods approach, using both quantitative and qualitative data. These were subjected to quality appraisal using both tools described above. The quality ratings for each study (strong, moderate, weak) are presented in the evidence tables (see Supplementary Materials).

### Data Extraction and Analysis

Data were extracted and findings organized based on (1) the type of study design employed, (2) the populations included, (3) the type of MBI strategies delivered and (4) the outcomes assessed. This was done in order to help construct the narrative and compare between disparate interventions when considering effectiveness.

Results are presented according to the narrative synthesis method outlined by Petticrew and Roberts (2008), which allows findings from multiple studies to be summarized and explained by constructing the story (i.e. the narrative that emerges from reading, extracting from, quality appraising and reconsidering included studies). Petticrew and Roberts (2008) suggest that a narrative synthesis is generally best presented in words, the method also allows for some statistical evidence to be used in the construction of the narrative. Where possible, standardized effect sizes (ESs) (Cohen’s *d*) were calculates and categorized, as ES ≥ 0.2 = small, ES ≥ 0.5 = medium and ES ≥ 0.8 = large. For studies that did not present data convertible to standardized effect sizes, *p* values were reported, wherever possible.

## Results

**Fig. 1:**
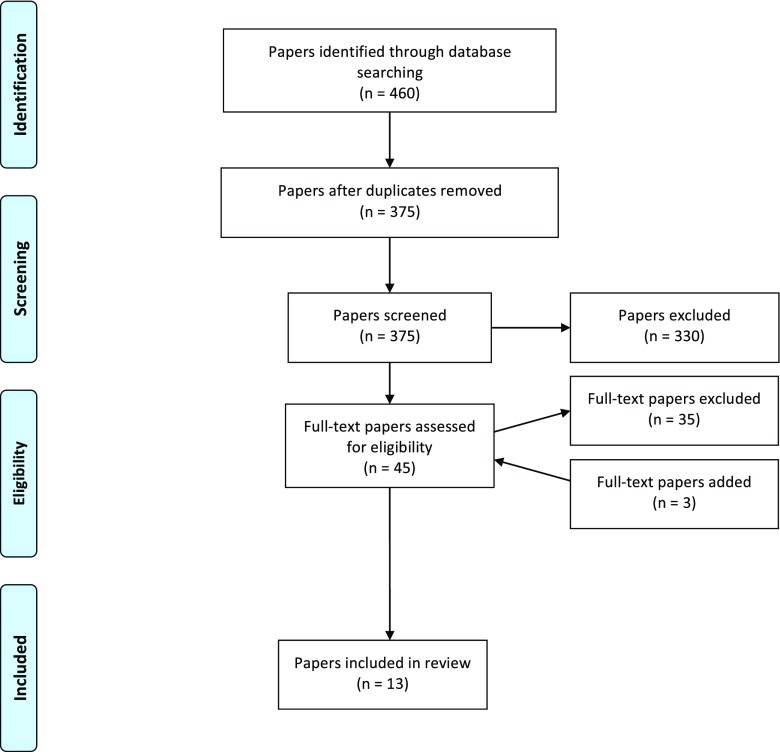
Flow diagram for paper screening results

Following de-duplication, the primary searches yielded 375 papers in total. After screening, 13 publications were included in the review (see Fig. 1). Eleven were considered as peer-reviewed journal publications, whilst two were not. One Indian study, published on the Vipassana Research Institute website (http://www.vridhamma.org), was considered not to have undergone independent peer review (Khurana and Dhar 2000), whilst another was an original PhD thesis and had not been published in a peer-reviewed journal (Flinton 1998).

### Study Characteristics

Table 1 provides an overview of the studies included in the review. The majority of studies were carried out in the USA (12/13; 92%). One study was conducted in India. Three (23%) were RCTs, one (8%) was a controlled trial (CT), three (23%) used a pre-post study design, three (23%) used a mixed-methods approach and three (23%) used a qualitative study design. One Indian study reported findings from a series of five disparate studies together, where different study designs had been used (CT or pre-post studies) in distinct populations. Where relevant, the results from each of these five studies are discussed. However, for convenience, this Indian study was placed in the “pre-post” category, as this was the method most commonly used.

**Table 1:** Study characteristics

Variables	Description
Country	USA (*n* = 12)
	India (*n* = 1)
Study design	Randomized controlled trial (RCT) (*n* = 3)
	Non-randomized controlled trial (CT) (*n* = 1)
	Pre-post (*n* = 3)^a^
	Mixed-methods (*n* = 3)
	Qualitative (*n* = 3)
Population	Male adolescent offenders (*n* = 11)
	Mixed gender: male adolescent and female adult (*n* = 1)
	Mixed gender: male adolescent and female adolescent (*n* = 1)
Intervention type	Mind body awareness (MBA) (*n* = 3)
	Structured mindfulness meditation (*n* = 2)
	Mindfulness meditation and cognitive therapy (*n* = 2)
	Mindfulness-based substance use (MBSU) intervention (*n* = 1)
	Mindfulness via the Internet (*n* = 2)
	One-to-one mindfulness (*n* = 2)
	Vipassana (*n* = 1)
Outcome measured	Mental health (*n* = 13)
	Mindfulness (*n* = 11)
	Problematic behaviour (*n* = 9)
	Self-regulation and emotional states (*n* = 9)
	Quality of life and wellbeing (*n* = 5)
	Substance use (*n* = 5)
	Personality, social and relational attitudes (*n* = 5)

^a^One study reports on five disparate studies; the main study design used was pre-post

Sample size varied markedly between studies, ranging from 27 (Himelstein et al. 2015) to 264 (Leonard et al. 2013) among the RCTs, with 42 (Flinton 1998) participants in the CT, 32 (Himelstein et al. 2012a) to 232 (Khurana and Dhar 2000) among the pre-post studies, 29 (Barnert et al. 2014) to 61 (Evans-Chase 2015b) among the mixed-methods studies and 10 (Himelstein et al. 2014) to 32 (Himelstein 2011) among the solely qualitative studies. Table 2 provides an overview of the study details, populations, intervention type and main outcomes reported.

**Table 2:** Scoping review: study details, population, intervention type and main outcomes

No.	Study	Population	Intervention	Outcome
Randomized controlled trials				
1	Himelstein et al. (2015)	Male adolescents (*n* = 27, assigned either 1:1 mindfulness or TAU), housed at a juvenile detention camp	1:1	Treatment group showed significantly greater increases in quality of life (ES = 0.60, *p* < 0.05) and staff rating of good behaviour (*p* < 0.05). No difference was seen in self-regulation (ES = 0.25, *p* > 0.05), substance use (ES = 0.32, *p* > 0.05) and mindfulness (ES = 0.09, *p* > 0.05). Both groups showed significant improvements in decision making (ES = 0.46, *p* < 0.01)
2	Evans-Chase (2013)	Male adolescents (*n* = 59; 29 IBM, 30 control group) housed in a juvenile justice facility	IBM	The oldest age group (19–23) in the treatment group scored significantly higher in self-regulation ability (*p* < 0.05) of post-intervention, than their similarly matched control counterparts
3	Leonard et al. (2013)	Male adolescents (*n* = 264; 147 MBCT, 117 active control), delivered at an urban correctional complex	CBT/MM	Overall task performance on the attention network test degraded in all participants. However, the magnitude of degradation was significantly less for the treatment group (ES = 0.30, *p* < 0.01)
Non-randomized controlled trials				
4	Flinton (1998)	Male adolescents (*n* = 42; 23 SMP, 19 control), residing in a camp for juvenile offenders.	SMP	SMP group, compared to controls, showed significant improvements in mental health (anxiety: ES = 1.14, *p* < 0.05) and in internal locus of control (ES = 1.47, *p* < 0.05)
Pre-post studies				
5	Le and Proulx (2015)	Male and female adolescents (*n* = 33), housed at the youth correctional facility	MBA	MBA participants showed significant improvements in mental wellbeing (stress: ES = 1.00, *p* < 0.05). Although not significant, a positive trend towards improvement was observed for impulsivity (ES = 0.32, *p* > 0.05), self-regulation (ES = 0.29, *p* > 0.05) and mindfulness (ES = 0.40, *p* > 0.05)
6	Himelstein et al. (2012a)	Male adolescent (*n* = 32), housed at a juvenile correctional facility in California	MBA	Participants showed significant improvements in mental health (stress: ES = 0.42, *p* < 0.05) and self-regulation (ES = 0.60, *p* < 0.001). No significant difference found on self-reported mindfulness, although it did show a trend towards improvement (ES = 0.22, *p* > 0.05)
7	Khurana and Dhar (2000), India	Male adolescents (*n* = 232) and female adults (*n* = 30) incarcerated at Tihar jail	VM	A significant improvement was seen in subjective wellbeing (SWB) (*p* < 0.01) and a decrease in the level of criminal propensity (*p* < 0.01) for those who attended VM
Mixed-methods				
8	Evans-Chase (2015b)	Male adolescents (*n* = 61), housed in a juvenile justice facility	IBM	No significant difference found on self-reported mindfulness (ES = 0.12, *p* > 0.05). Participants described using class skills to regulate emotions and behaviour and deal with conflict in the facility
9	Barnert et al. (2014)	Male adolescents (*n* = 29), delivered at a juvenile correctional facility	MBA	Participants showed a significant increase in self-regulation (ES = 0.44, *p* < 0.05). Although showing a trend towards improvement, stress (ES = 0.32, *p* = 0.29), mindfulness (ES = 0.20, *p* = 0.31) and impulsivity (ES = 0.20, *p* = 0.30) did not reach statistical significance. The young men spoke about feeling better, feeling more in control, improved self-awareness and increased social cohesiveness. They also spoke about a resistance to the meditation practice and future use of the techniques
10	Himelstein (2011)	Male adolescents (*n* = 48), housed at a juvenile correctional facility	MBSU	The results showed a significant decrease in impulsivity (ES = 0.43, *p* < 0.01) and a significant increase in perceived risk of drug use (ES = 0.75, *p* < 0.05) from pre-test to post-test. No significant difference was found on self-regulation, although a trend towards improvement was shown (ES = 0.25, *p* > 0.05)
				From the focus group discussions, 3 major themes were identified: receptivity to the program in general, appreciation of the facilitators teaching style and learning about drugs
Observational; semi-structured interviews				
11	Himelstein et al. (2015)	Male adolescents (*n* = 10), housed in a Californian detention centre	1:1	The main themes identified were as follows: enhanced psychological mindfulness and wellbeing, development of worldview, novel experiences, challenging experiences and future use
12	Himelstein et al. (2012b)	Male adolescents (*n* = 32), housed at a juvenile correctional facility in California	MBA	Main themes identified were as follows: increased wellbeing, improved self-regulation, increased awareness and an accepting attitude towards the intervention
13	Derezotes (2000)	Male adolescent sex offenders; it is unclear how many participants were used in this study	SMP	The participants reported feeling more relaxed, having improved concentration, improved impulse control and being less disturbed by thoughts. Being treated with respect, care and humanness was also identified as important. Parents and facilitators were supportive of the course

### Populations, Attrition and Follow-Up

Twelve (92%) of the 13 studies focused on incarcerated young males. One study included both incarcerated young males and females. Across the studies, there were approximately 842 participants (exact participant numbers in one study were unclear; Derezotes 2000). Of these 842 participants, 833 (99%) were male adolescents and nine (1%) were female adolescents. Ages of the included participants varied, ranging from 14 to 23 years. Ethnicity was poorly characterized; where reported (*n* = 6 studies), most participants were Black or Latino (98%) (Leonard et al. 2013), Latino (range 59–75%) (Barnert et al. 2014; Himelstein 2011; Himelstein et al. 2012a, 2015) or Hawaiian (60%) (Le & Proulx, 2015). Most participants were housed in a juvenile correctional facility, with one facility being a community-based human service agency for adolescent sex offenders (see Table 2).

Attrition was defined differently between studies, as either those participants who did not complete the intervention or those participants who did not complete the outcome measures. Attrition rates for those who did not complete the intervention varied, and only half of the quantitative studies reported these data (5/10; 50%) (see Table 3). None of the included studies defined intervention completion. Overall, attrition from the intervention ranged from 8 to 40%, with a mean (SD) value of 22% (13.5). Intervention attrition percentages and reasons accounting for these are detailed in Table 3.

**Table 3:** Attrition rates as defined by intervention completion

Study (country)	Study design	Intervention (sample size)	Non-completers (%)	Reason (*n*)
Le and Proulx (2015) (USA)	Pre, post	MBA (*n* = 36)	8	Lost interest (2)
Barnert et al. (2014) (USA)	CT	MBA (n = 29)	10	Released (6)
Himelstein (2011) (USA)	Pre, post	MBSU (*n* = 60)	20	Released (12)
Flinton (1998) (USA)	CT	SMP (*n* = 62)	30	Administrative issues (16)*
				Released (4)
Himelstein et al. (2012a) (USA)	Pre, post	MBA (*n* = 47)	40	Released (15)

Study design: randomized controlled trial (RCT) and non-randomized controlled trial (CT)

Interventions used: mindfulness-based stress reduction (MBSR) and mind body awareness (MBA)

*MBSU* mindfulness-based substance use, *SMP* structured meditation program

*A whole cohort was removed from the study as one of the camps failed to adhere to the study requirements, removing participants from the group and adding new members who had not completed baseline measures

Three (30%) RCTs (Evans-Chase 2015a; Himelstein et al. 2015; Leonard et al. 2013) provided details for attrition in terms of data collection. Attrition rates for those who did not complete outcome measures varied. Overall, attrition from data collection ranged from 25 to 56%, with a mean (SD) value of 40% (15.5). Data collection attrition percentages and reasons accounting for these are detailed in Table 4.

**Table 4:** Attrition rates as defined by data collection

Study (country)	Study design	Intervention (sample size)	Attrition rate (%)	Reason (*n*)
Leonard et al. (2013) (USA)	RCT	MM-CBT	25^*^	Transfer/release (20%)
				Corrupt computer files (3%)
				Refusal (1.5%)
				Deportation (0.5%)
Himelstein et al. (2015) (USA)	RCT	I:I	39	Released (21%)
				Incomplete measures (18%)
Evans-Chase (2013) (USA)	RCT	IBM	56	Release from custody (31%)
				Withdrawal from the study (13%)
				On lockdown (5.5%)
				Removed from analysis (5.5%)
				Incomplete measures (1%)

Study design: randomized controlled trial (RCT)

Interventions used: Internet-based mindfulness (IBM), one-to-one (1:1) mindfulness, mindfulness meditation and cognitive behavioural therapy (MM-CBT)

*This figure represents attrition at 15 weeks of follow-up. Attrition immediately post intervention was 42%

### Quality Appraisal

All of the quantitative studies had methodological limitations, including unclear recruitment techniques, small sample sizes, high attrition rates, failure to control for important confounders, use of non-validated measures and inconsistent reporting. Seven of the 10 papers that presented quantitative data were assigned a score of moderate, and three were assigned a weak score. None received a score of strong. Studies scored moderately on potential for blinding (9/10), study design (6/10), selection bias (5/10), confounders (5/10) and descriptions of withdrawal and dropout (5/10). The category where studies scored most strongly was data collection methods (7/10) (see Supplementary Materials). Quality scoring of the qualitative studies found scores of weak for one study and moderate for five studies. None of the qualitative studies were allocated a score of strong (see Supplementary Materials).

### Interventions

The “Template for Intervention Description and Replication” (TIDieR) checklist and guide was referred to when extracting data about the interventions used in the included studies (Hoffmann et al. 2014). All interventions included at least two of the three core MBSR techniques (i.e. breath awareness, body awareness and/or mindful movement). The interventions were characterized as mind body awareness (MBA), structured meditation program (SMP), Vipassana meditation (VM), cognitive behavioural therapy and mindfulness meditation (CBT/MM), mindfulness-based substance use (MBSU), Internet-based mindfulness (IBM) and one-to-one (1:1) mindfulness. See supplementary materials for a more detailed description of the categorized interventions.

Interventions varied in terms of (1) setting, (2) content, (3) dose and duration, (4) format, (5) teacher characteristics and (6) institutional constraints. In terms of setting, the quality and conditions of space provided varied widely, with some facilities being modified to suit the needs of the meditation course. For example, in one study, a Vipassana centre was set up to create a “live-in” meditation hall with areas for sleeping and eating (Khurana and Dhar 2000). Other courses were delivered in a less tailored setting (i.e. dormitories in which the young men resided).

In terms of content, three interventions had a mindfulness-based curriculum specifically adapted for incarcerated young men including psychotherapeutic topics relevant to their specific needs, mindfulness meditation, experiential activities and discussion time (Barnert et al. 2014; Himelstein et al. 2012a, 2012b). One study was tailored towards drug rehabilitation (Himelstein 2011). Another study merged mindful meditations with relaxation techniques—Jacobson’s progressive muscle relaxation technique (Flinton 1998). One was heavily influenced by Buddhist teachings (Khurana and Dhar 2000). One study integrated social cognitive change components of CBT/MM (Leonard et al. 2013). One was delivered via the Internet (Evans-Chase 2015b) and another on a one-to-one basis, where mindfulness meditations were coupled with motivational interviewing, goal planning and preparatory work towards re-entry back into the community (Himelstein et al. 2015).

Dose and duration ranged from 1 to 10 h per session, being delivered daily over a period of 10 days, or weekly over a period of 3 to 12 weeks (1–2 h daily). Some were delivered bi-weekly. One study included an intensive day package as an adjunct to the 10-week course (Barnert et al. 2014). The VM course was delivered as an intensive silent retreat where participants spent up to 10 h per day in meditation and were provided with daily teaching on Buddhist philosophy, followed a vegetarian diet, and were separated from the rest of the incarcerated population and from social contacts. The mindfulness-based approaches were more secular by comparison and were instead integrated into daily prison routines, with classes taking place weekly or bi-weekly, over 1 to 2 h, more closely adhering to the MBSR-type protocol.

Four studies did not include teacher characteristics (Barnert et al. 2014; Derezotes 2000; Flinton 1998; Khurana and Dhar 2000). Of those that did, details were generally vague. One study was delivered online using MP3 downloads, and the paper provided the name of the teacher and referred readers to his website (Evans-Chase 2015b). Only one study provided details of the teachers’ own meditation practices, which ranged from 5 to 30 years (Le & Proulx, 2015). Four courses were delivered by clinical psychologists, clinicians or social service workers who had both psychotherapeutic and meditation skills (Himelstein 2011; Himelstein et al. 2012a; Le & Proulx, 2015; Leonard et al. 2013). Only one study referred to clinical supervision being provided (Leonard et al. 2013). The number of teachers delivering the courses varied. In one study, the author assumed the role of both researcher and teacher, raising the risk of researcher bias (Himelstein et al. 2012a).

Various authors noted that administrative, organizational and institutional constraints, such as shared cells and other restrictions of prison life, limited full participation in the interventions. For example, space, privacy and noise constraints limited participants’ ability to practise the meditation exercises in a number of settings. In some studies, resources were sparse or limited, with no provision of homework material allowed. In one study, recording of homework adherence was curtailed due to a violent incident resulting in the confiscation of pens from all participants (Himelstein 2011).

### Outcomes

Studies reported on a wide range of outcomes, mainly from self-report questionnaires. One study used an objective physiological measure (Le & Proulx, 2015), and two included behavioural measures (i.e. behavioural regulation data collected via third person observations and ratings assigned by detention staff members) (Barnert et al. 2014; Himelstein et al. 2015). One study used a computerized attention network test (ANT) to index attentional task performance (Leonard et al. 2013). No adverse events were reported in any of the included studies.

### Quantitative Outcome Findings

The full range of outcome assessments numbered 17 (see Supplementary Materials). For pragmatic reasons, we have classified these as follows: self-regulation and emotional states (*n* = 8), mindfulness (*n* = 5), mental health (anxiety and stress) (*n* = 4), problematic behaviour (impulsivity) (*n* = 3), QOL and wellbeing (*n* = 2), substance use (*n* = 2) and criminal propensity (*n* = 1). Follow-up generally took place immediately after the intervention only. One study collected additional data at four months post baseline (Leonard et al. 2013). The following section describes these outcomes in relation to the type of studies in which they were used.

Eight of the ten quantitative studies measured self-regulation and emotional states. Five reported significant improvements in emotional stability and self-regulation ability. These data are based upon three RCTs (Evans-Chase 2013; Himelstein et al. 2015; Leonard et al. 2013), one CT (Flinton 1998) and four pre-post study designs (Barnert et al. 2014; Himelstein 2011; Himelstein et al. 2012a; Le & Proulx, 2015). One RCT (*n* = 61) found that older participants (ages 19–23) who received IBM scored higher on interpersonal self-restraint (Restraint-Weinberger Adjustment Inventory) compared to controls (*p* < 0.05) (Evans-Chase 2013). Another RCT (*n* = 147) reported a significantly lower degradation in performance on the ANT for those who had attended CBT/MM compared to the active control group (ES = 0.30, *p* < 0.01). For those in the CBT/MM group, performance remained stable over time among those who practised outside of the teaching sessions compared to those who did not (Leonard et al. 2013). Two studies examined prisoners’ perception of control using the Prison Locus of Control Scale (PLCS). In a small RCT (*n* = 27), Himelstein et al. (2015) reported no significant difference in the incarcerated young men’s perception of control compared to the control group (ES = 0.25, *p* > 0.05) (Himelstein et al. 2015). In contrast, a small (*n* = 42) CT delivering SMP reported significant improvements in participants’ internal locus of control (ES = 1.47, *p* < 0.05) (Flinton 1998). Four pre-post studies (Barnert et al. 2014; Himelstein 2011; Himelstein et al. 2012a; Le & Proulx, 2015) delivering MBA reported contrasting results using the Healthy Self-Regulation Scale (HRS) measure. Two studies (Barnert et al. 2014; Himelstein et al. 2012a) (*n* = 32 and *n* = 29) demonstrated a significant increase in self-regulation ability (ES = 0.44, *p* = 0.01; ES = 0.60, *p* < 0.01), whilst the other two (Himelstein 2011; Le & Proulx, 2015) (*n* = 48 and *n* = 33) reported no significant difference (ES = 0.25, *p* > 0.05; ES = 0.29, *p* > 0.05).

Five studies reported on levels of mindfulness following training. These included two RCTs (Evans-Chase 2015a; Himelstein et al. 2015), one CT (Barnert et al. 2014) and two pre-post studies (Himelstein et al. 2012a; Le & Proulx, 2015). These studies delivered MBA (*n* = 3), 1:1 mindfulness and IBM. No significant changes were found.

Three of four studies using mental wellbeing as an outcome reported benefit. A CT (*n* = 42) delivering SMP reported significant reductions in measures of anxiety using the Brief Symptom Inventory (BSI) (ES = 1.14, *p* < 0.05), whilst two pre-post studies (*n* = 32; *n* = 33) delivering MBA reported significant reductions in levels of perceived stress (Perceived Stress Scale (PSS); ES = 0.42, *p* < 0.05; ES = 1.00, *p* < 0.05) (Himelstein et al. 2012a; Le & Proulx, 2015).

Quantitative measures of problematic behaviour were reported in three pre-post studies. All three measured impulsiveness in incarcerated male adolescents using the Teen Conflict Survey (TCS). Himelstein (2011; *n* = 48) reported significant reductions post MBSU (ES = 0.43, *p* < 0.01). In contrast, Le and Proulx (2015; *n* = 33) and Barnert et al. (2014; *n* = 29) reported no significant changes in impulsivity among adolescents receiving MBA (ES = 0.32, *p* > 0.05; ES = 0.20, *p* = 0.20). In addition, Himelstein et al. (2015) showed significantly lower infractions on behavioural points, as documented by juvenile detention centre officials.

Two of the ten quantitative studies reported on QOL. A RCT used the Rosenberg Self-Esteem Scale (RSE) and demonstrated significant improvements post 1:1 mindfulness (ES = 0.60, *p* < 0.05) (Himelstein et al. 2015). A pre-post study demonstrated significant improvements in QOL (*p* < 0.01) using the Subjective Wellbeing Scale (SWS) (Khurana and Dhar 2000).

Two of the ten studies reported on substance use. A pre-post study (*n* = 48) using the Monitoring the Future (MTF) questionnaire reported a significant increase in awareness of drug use risk among incarcerated male adolescents (ES = 0.75, *p* < 0.05) (Himelstein 2011). However, a RCT (*n* = 35) reported no significant difference on the MTF following 1:1 mindfulness versus control (ES = 0.32, *p* > 0.05) (Himelstein et al., 2015).

One pre-post study reported on criminal propensity, finding a significant decrease in criminal propensity in adolescent males versus matched controls following VM training (*p* < 0.01) (Khurana and Dhar 2000).

### Qualitative Findings

Qualitative findings are based on participants' collective experience of a variety of different interventions: SMP, MBA, IBM and MBSU. General themes revolved around two key areas. The first was internal changes, such as participants feeling more relaxed, better able to manage stress, better self-regulatory skills, improved self-awareness and being more optimistic about future prospects. The second related to external changes, such as improved relationships, valuing kindness shown by the teacher and being part of a supportive environment in which they felt respected and valued. Participants also reported feeling empowered by having met the challenge involved in adhering to the mindfulness practices. They appreciated being part of the group; developed more positive relationships with staff, peers and family; felt more in control; and were better able to cope with difficult feelings and impulses. They especially appreciated being treated with care, respect and humaneness. In the main, participants, family and staff were enthusiastic about and supportive of the courses. However, two studies highlighted small numbers of participants being resistant to the meditative practices (Barnert et al. 2014; Himelstein et al. 2015).

## Discussion

This scoping review evaluated the existing evidence for MBIs among young offenders. It demonstrated that the existing international evidence regarding the utility of MBIs in youth offending populations is limited and the optimal approach unknown. Three RCTs, one CT, three pre-post, three mixed-methods and three qualitative studies were included in the review. Pooled numbers produced an overall number of 842 participants, mainly male (only 9/842 participants were female). Where reported, participant ages ranged from 14 to 23. In general, ethnicity was poorly characterized and the quality of methods was a limiting factor in almost all included studies. A wide range of MBIs was used, and thus, it was not possible to identify an optimal approach in terms of content, dose or intensity. Quantitative outcome measures were diverse, covering different aspects of mental health and wellbeing. Where applicable, effect sizes ranged widely. From the six studies that collected qualitative data, commonly reported benefits revolved around two key areas: internal changes (such as feeling less stressed, better able to manage difficult emotions) and external changes (such as improved relationships). Three important future considerations have been highlighted via the findings derived from this scoping review: (1) there is limited research regarding the use of MBIs among young female offenders, (2) no significant changes were demonstrated in levels of mindfulness (in either direction) following training and (3) there are multiple and complex challenges associated with researching MBIs within the prison setting.

Findings from this scoping review resonate with those reported by Shonin et al. (2013) in a systematic review of Buddhist-derived interventions (BDIs) in correctional institutions. They reported recurrent issues with the quality of study methods, as did a more recent systematic review investigating yoga and meditation in offending populations (Auty et al. 2015). This current review found that three RCTs have been conducted, only two being of strong methodological quality, one of which was an unpublished PhD thesis, overlooked by all preexisting reviews. Shonin et al. (2013) reported only two RCTs, whereas Auty et al. (2015) found four, but these were mixed with yoga interventions.

The current review demonstrated discrepancies in intervention delivery, content and outcomes, which makes it difficult to draw meaningful comparisons between interventions across studies. This is in keeping with Shonin et al. (2013), where meta-analysis was deemed impossible due to the high levels of heterogeneity among interventions and outcome measures.

Future research could potentially explore whether MBSR can be delivered in its standardized format or whether the intervention needs to be adapted to meet the specific needs and issues faced by young people who offend. Although meta-analysis was not possible in this scoping review, examination of discernable effect sizes suggests a broad range of potential effectiveness across diverse interventions and outcomes. This could imply that by practising the core components of mindfulness, young offenders may experience improvement in psychological and emotional wellbeing and in behavioural functioning. Shonin et al. (2013) did not report effect sizes in their systematic review. However, Auty et al. (2015) did, demonstrating small beneficial effects on psychological wellbeing (ES = 0.46) and behavioural functioning (ES = 0.30). Auty et al. (2015) also showed that longer duration and less intense interventions were associated with larger effects.

### Strengths and Limitations

The scoping review approach allowed a diversity of study methods to be assessed along with assessment of study quality, augmenting interpretation of results. However, for the qualitative studies, the research team applied a quality rating scale that they devised themselves. Such an approach might obscure rather than enhance interpretation. For example, it does not consider “weighting” among the 18 assessment criteria where some markers of quality may be more important than others. In addition, using two different appraisal methods makes it difficult to compare the relative value of quantitative studies compared to qualitative studies.

Publication bias is a major threat to the validity of any review. Therefore, obtaining and including data from unpublished trials is one way of addressing this bias. The possibility of not identifying relevant papers is a potential limitation. However, to counter this, a comprehensive search strategy was used, which included the grey literature (e.g., one relevant unpublished PhD thesis was identified and included). In addition, only English language studies were included, which may have resulted in the omission of relevant empirical findings.

The lack of a consensus definition of mindfulness is a source of ambiguity in clinical and research domains (Khoury et al. 2017). The current review sought to include studies defining MBIs in similar terms to Kabat-Zinn (i.e. including core components). Other interventions that draw upon mindfulness, such as acceptance and commitment therapy (ACT) and dialectical behavioural therapy (DBT), were not included. These approaches, along with other related interventions (Yoga, Tai-Chi, compassion-focused therapy, loving kindness meditation), may also have a potential role in youth offending settings.

### Research Direction and Implications for Practitioners

Young females are underrepresented in the research and feasibility of MBIs within this population unclear. In this review, only 1% of the populations studied were adolescent females. This may reflect the low prevalence of females within the criminal justice system in general. Demographic figures from the Federal Bureau of Prisons report a 6.8% prevalence of female offenders compared with a 93.2% prevalence of male offenders (Federal Bureau of Prisons 2017). Data from the UK suggests that female prisoners have a greater prevalence of mental health complaints, which might make them more likely to seek out interventions. However, they are also reported to have unique needs when compared to male counterparts (less impulsivity and hostility; higher substance use and psychosis) rendering direct comparison a challenge (Birmingham 2003). True prevalence of mental illness remains unknown in this context, and mental health concerns are likely to be undetected and untreated in offending populations as a whole (Birmingham 2003).

At present, comparing MBI studies among young people who offend is challenging due to considerable heterogeneity in terms of study design, populations, interventions and outcome measures used. In addition, mindfulness is potentially an ambiguous term, where a lack of a consensus definition, standardized intervention and/or specific outcome measure for mindfulness in this context makes it difficult to assess fidelity and effectiveness. What mindfulness means to young offenders, how it should be delivered and if it can address their complex needs remain to be convincingly established. Active MBI ingredients remain to be subject to review (Crane et al. 2017; Gu et al. 2015), further compounding difficulties in creating the optimal approach for this particular population. Existing studies among youth offending populations have generally not recorded treatment adherence, a key factor in intervention effectiveness in other populations (Parsons et al. 2017). Therefore, although mindfulness is thought to be a key mediator of beneficial outcomes from MBIs (Alsubaie et al. 2017), measuring it as an outcome among youth offending populations is challenging.

A goal of future studies may be to establish agreed definitions and create standardized protocols (Crane et al. 2017; Dimidjian and Segal 2015). Such clarity could greatly enhance interpretation of study findings, provide a standardized platform from which future researchers can build and ensure MBI teachers are appropriately trained and that the young people are indeed receiving mindfulness training.

Using standardized outcome measures may also facilitate comparison. Future controlled studies are required, ideally RCTs powered to detect definitive evidence of effectiveness. Little remains known about how these interventions compare with other commonly used psychological interventions, such as CBT, or how cost-effective they might be.

Further high quality studies are needed if feasibility, effectiveness and implementability of these interventions are to be established. There is an urgent need for rigorous primary evaluations of mindfulness interventions for reducing factors known to increase the risk of re-offending, to develop this field and to guide future youth offending prevention programs and policies. In order to provide effective, evidence-based care for both male and female young offenders, it is essential to conduct research with this population to reflect their particular circumstances and complex needs (Wakai et al. 2009). However, the challenge of conducting rigorous research within a prison setting must also be acknowledged and accommodated for in future research in this area.

Researchers working in this setting need to anticipate potential organizational and/or logistical issues that may delay or disrupt the research process (Byrne 2017), for example background checks, compulsory training and seeking permission to bring research materials onsite (Wakai et al. 2009). Retaining research participants is a concern for all researchers, but incarcerated populations have an exceptionally high attrition rate for a number of reasons (e.g., transfer, release, participants on remand, court appearance, medical visits, family visits, administrative segregation), which can often be unanticipated (Byrne 2017; Shonin et al. 2013; Wakai et al. 2009). Researcher need to work collaboratively with prison staff and plan well in advance so that necessary procedures can be put in place to minimize the burden to prison staff and movement of the young offenders. Developing effective interventions to serve this population is paramount, and research approaches need to be responsive to the challenges faced in this type of setting as well as gaining a better understanding as to what these young people need. Therefore, there is a need for mixed-methods research designs, especially if RCTs are not implementable in real-life settings.

## Electronic supplementary material

ESM 1 (DOCX 46 kb)
