# Supplementary material for: Mindfulness-Based Interventions for Young Offenders: a Scoping Review
Source: Mindfulness (N Y). 2018 Feb 21;9(5):1330–43. doi: 10.1007/s12671-018-0892-5 (PMC6153893; doi:10.1007/s12671-018-0892-5)
Supplement: Supplementary file 1 — (DOCX 46 kb) [file 12671_2018_892_MOESM1_ESM.docx]

1: Quality scores for the quantitative studies included in this review

| **No** | **Study** | **Selection Bias** | **Study Design** | **Confounders** | **blinding** | **Data Collection Methods** | **Withdrawal and dropout** | **Overall Rating** |
| --- | --- | --- | --- | --- | --- | --- | --- | --- |
| 1 | Himelstein (2015) | 2 | 1 | 1 | 2 | 1 | 2 | **2 (Moderate)** |
| 2 | Le & Proulx (2015) | 2 | 2 | 2 | 2 | 1 | 2 | **2 (Moderate)** |
| 3 | Barnert et al. (2014) | 3 | 2 | 1 | 2 | 2 | 1 | **2 (Moderate)** |
| 4 | Evans-Chase (2015) | 2 | 1 | 1 | 2 | 1 | 3 | **2 (Moderate)** |
| 5 | Evans-Chase (2013) | 2 | 1 | 1 | 2 | 1 | 3 | **2 (Moderate)** |
| 6 | Leonard et al (2013) | 1 | 1 | 2 | 2 | 1 | 2 | **2 (Moderate)** |
| 7 | Himelstein et al. (2012) | 3 | 2 | 2 | 3 | 2 | 2 | **3 (Weak)** |
| 8 | Himelstein et al.(2011) | 2 | 2 | 2 | 2 | 2 | 1 | **2 (Moderate)** |
| 9 | Khurana & Dhar (2000) | 3 | 2 | 2 | 2 | 1 | 3 | **3 (Weak)** |
| 10 | Flinton (1998) | 3 | 2 | 3 | 2 | 1 | 2 | **3 (Weak)** |
|  | **Total for a score of 1** | **1** | **4** | **4** | **0** | **7** | **2** | **19** |
|  | **Total for a score of 2** | **5** | **6** | **5** | **9** | **3** | **5** | **38** |
|  | **Total for a score of 3** | **4** | **0** | **1** | **1** | **0** | **3** | **9** |

2: Quality scores for the qualitative studies included in this review

| No. | Study | Intervention | Quality Score |
| --- | --- | --- | --- |
| 1 | Evans-Chase (2015) (Mixed Methods) | IBM | Moderate |
| 3 | Himelstein et al (2015) | 1:1 | Moderate |
| 4 | Barnert et al. (2014) (Mixed Methods) | MBA | Moderate |
| 6 | Himelstein et al. (2012) | MBA | Moderate |
| 7 | Himelstein et al. (2011) (Mixed Methods) | MBSU | Moderate |
| 9 | Derezotes (2000) | SMP | Weak |

3: Included interventions characterized by the type of therapy or skills they were teaching

| Intervention | Description | Studies that used this approach |
| --- | --- | --- |
| Mind Body Awareness (MBA) | Group based intervention specifically tailored to the diverse needs of incarcerated youths.  Delivered over a duration of 10-weeks, lasting 90 minutes.  Weekly session includes:   - Check-in (i.e. sharing present moment feelings) - Specific experiential emotional-intelligence activities (60 minutes) - Formal meditation (30 minutes)   Strong emphasis on the clinical psychotherapy component, which covers the following topics; (1) goodness, (2) mindfulness, (3) active listening, (4) impulse regulation, (5) emotional-intelligence, (6) empathy, (7) forgiveness, (8) transforming negative core beliefs, (9) cause and effect, and (10) interpersonal relationships. All sessions end with a dedication of ‘positivity’ | Barnert et al. (2014)  Himelstein et al. (2011)  Le & Proulx (2015) |
| Structured Meditation Programme (SMP) | These interventions contained techniques such as:   - Breath-awareness practices - Mindfulness of sound - Walking meditation - Moving meditation (Hatha Yoga) - Loving kindness or compassion meditations.   Basic instructions are provided, including guidance on meditation techniques. Discussions, reflections, and group sharing of experiences contributed to the overall content of intervention. | Derezotes (2000)  Flinton (1998) |
| Vipassana Meditation (VM) | Intensive 10-day, silent residential.  Daily schedule consists of:   - 8- 10 hours of sitting meditation, interspersed with regular break, - Teachings on Buddhist principles.   Participants are required to follow five precepts; including abstention from: (1) killing; (2) stealing; (3) sexual activity; (4) lying; and (5) taking intoxicants. All participants are required to follow a vegetarian diet. | Khurana & Dhar (2000) |
| Intervention | **Description** | **Studies that used this approach** |
| Cognitive Behavioural Therapy and Mindfulness Meditation (CBT/MM) | Based on the integration of cognitive behavioural techniques and mindfulness meditation. Adapted to suit needs of prison population. Delivered over a duration of 3-6 weeks, lasting 1.5 - 2.5 hours, typically comprising of 8 -12 participants (No day retreat included).  The programme consists of three core mindfulness practices   - Body scan, - Sitting meditation, - Mindful movement (walking meditation/yoga)   Time was allocated for group discussion. After each group session, participants were assigned daily homework exercises. | Leonard et al. (2015) |
| Mindfulness-Based Substance Use (MBSU) | This intervention is specifically orientated towards drug education and the development of self-awareness.  Typically delivered over a period of 8 weeks, lasting 1.5 hours.  Sessions include:   - ‘Mindful’ check-in; - Experiential group activities (including mindfulness); - Group discussion; - Didactic training.   Drug education activities include: (a) drug category awareness; (b) potential implications of mixing certain drugs; (c) positive and negative aspects of drugs and; (d) physiological effects of drug use.  Didactic aspects are used to open up discussion and as a platform to unpack personal experiences and develop self-awareness. | Himelstein (2011) |
| Internet-based Mindfulness (IBM) | Treatment sessions based on recordings of lectures and guided meditations by Noah Levine, a Budhist practitioner. Audio recordings are available for free from his website http://www.dharma- punx.com/htm/mp3.htm.  Sessions began with a lecture-like presentation of mindfulness, influenced by Buddhist teachings, covering the following topics:   - Compassion, - Patience, - Mindfulness in everyday life   These presentations were followed by guided meditation encouraging participants to focus on the breath, assuming an in a nonjudgmental stance towards any thoughts and feelings arising. | Evans-Chase (2013; 2015) |
| Intervention | **Description** | **Studies that used this approach** |
| One-to-one mindfulness (1:1) | Mindfulness meditation training delivered alongside individual counselling. Meditation time ranged from 5 to 25 minutes per session. These practices were coupled with motivational interviewing, goal planning, and successful re-entry back into the community.  Specific, formal, meditations were taught over the first six weeks. These included:   - Deep breathing - Mindfulness of the breath - The body-scan - A non-moving body scan - Counting meditation   Informal meditations were also taught (Stop, Take a breath, Imagine the consequences, Chose a response- STIC). Following training in these practices participants were then encouraged to chose the meditation they naturally gravitated towards, based on their own individual needs. Participants were encouraged to practice the techniques they were taught between sessions, | Himelstein (2015) |

4: Outcome measures used in the studies included in this review

| Outcomes | Measures Used | Study | N |
| --- | --- | --- | --- |
| *Self-regulation and emotional states* | Attention Network Test (ANT; Fan et al., 2002)  Healthy Self-Regulation Scale (HSR; West, 2008)  Restraint-Weinberger Adjustment Inventory (RWAI; Weinberger, 1996)  Prison Locus of Control Scale (PLOCS; Pugh, 1994, 1992)  Decision-making Skills (Hanson, 1996) | Barnert et al. (2014); Evans-Chase (2013); Flinton (1997);Himelstein et al. (2015); Himelstein et al. (2011); Himelstein (2011); Le & Proulx (2015) ; Leonard et al (2013) | 8 |
| *Mindfulness* | Mindfulness Attention Awareness Scale (MAAS; Brown & Ryan, 2003)  Mindfulness Attention Awareness Scale- Adolescent version (MAAS -A; Brown, West, Loverich & Biegel, 2011)  5-Facet Mindfulness Questionnaire (FFMQ; Baer et al. 2008)  Child and Adolescent Mindfulness Measure (CAMM; Greco, baer, & Smith, 2011) | Barnert et al. (2014); Evans-Chase (2015); Himelstein et al. (2015); Himelstein et al. (2011); Le & Proulx (2015) | 5 |
| *Mental health* | Perceived Stress Scale (PSS; Cohen et al., 1983)  Brief Symptom Inventory (BSI; Derogatis & Spencer, 1982) | Barnert et al. (2014); Flinton (1997); Himelstein et al. (2011); Le & Proulx (2015) | 4 |
| *Problematic behavior* | Teen Conflict Survey-Impulsiveness Subscale (TCS; Bosworth & Espelage, 1995) | Barnert et al. (2014); Himelstein (2011); Le & Proulx (2015) | 3 |
| *Quality of Life and Wellbeing* | Subjective well-being Inventory (SWBI; Nagpal & Sell, 1985)  Life Satisfaction Scale (PGI; Chandigarh, 1986)  Rosenberg Self-Esteem Scale (SES; Rosenberg, 1979) | Himelstein et al. (2015); Khurana & Dhar (2000) | 2 |
| *Substance use* | Monitoring the future questionnaire (MTF; Johnston et al., 1991) | Himelstein et al. (2015); Himelstein (2011); | 2 |
|  |  |  |  |
| *Criminal Propensity* | Criminal Propensity Scale (Sanyal & Kathpalia, 1999) 0 | Khurana & Dhar (2000) | 1 |
